# Unusual behaviour of phototrophic picoplankton in turbid waters

**DOI:** 10.1371/journal.pone.0174316

**Published:** 2017-03-27

**Authors:** Boglárka Somogyi, Károly Pálffy, Katalin V. -Balogh, Zoltán Botta-Dukát, Lajos Vörös

**Affiliations:** 1 Department of Hydrobotany, Balaton Limnological Institute, MTA Centre for Ecological Research, Tihany, Hungary; 2 Department of Terrestrial Ecology, Institute of Ecology and Botany, MTA Centre for Ecological Research, Vácrátót, Hungary; Universidade Federal do Estado do Rio de Janeiro, BRAZIL

## Abstract

Autotrophic picoplankton (APP) abundance and contribution to phytoplankton biomass was studied in Hungarian shallow lakes to test the effect of inorganic turbidity determining the size distribution of the phytoplankton. The studied lakes displayed wide turbidity (TSS: 4–2250 mg l^-1^) and phytoplankton biomass (chlorophyll *a*: 1–460 μg l^-1^) range, as well as APP abundance (0 and 100 million cells ml^-1^) and contribution (0–100%) to total phytoplankton biomass. Inorganic turbidity had a significant effect on the abundance and contribution of APP, resulting in higher values compared to other freshwater lakes with the same phytoplankton biomass. Our analysis has provided empirical evidence for a switching point (50 mg l^-1^ inorganic turbidity), above which turbidity is the key factor causing APP predominance regardless of phytoplankton biomass in shallow turbid lakes. Our results have shown that turbid shallow lakes are unique waters, where the formerly and widely accepted model (decreasing APP contribution with increasing phytoplankton biomass) is not applicable. We hypothesize that this unusual behaviour of APP in turbid waters is a result of either diminished underwater light intensity or a reduced grazing pressure due to high inorganic turbidity.

## Introduction

Autotrophic picoplankton (APP), which comprises small (<2 μm) prokaryotic picocyanobacteria and eukaryotic phototrophs, is of great importance in the carbon cycling of oceans and lakes [1,2]. Its widespread incidence was discovered in the late seventies and early eighties in several parts of the world [3–5]. APP is a major component of the photosynthetic biomass in many aquatic ecosystems, particularly in oligotrophic lakes and oceans [6,7].

Thus, it constitutes an important source of energy in aquatic food webs as an integral part of the microbial loop [2]. The occurrence and dynamics of APP are influenced by several environmental factors, such as light intensity, water temperature, salinity, nutrient supply, grazing and viral infection [2,8,9]. It is widely accepted that the absolute importance of APP (abundance and biomass) increases while its relative importance (their percentage in total biomass and primary production) decreases with increasing trophic status [7–10]. Watson and McCauley [11] explained the low contribution of APP in nutrient-rich (eutrophic) environments by a higher grazing pressure on APP than on larger-sized phytoplankton. In oligotrophic environments, however, APP cells have more benefits due to their tiny size and larger surface/volume ratio resulting in more efficient nutrient uptake [12].

The above observations were confirmed by the quantitative regression models of Bell and Kalff [13], which described the relative and absolute importance of picoplankton based on the phytoplankton biomass (chlorophyll *a*) in freshwater and marine ecosystems. As an exception to a rule, the contribution of APP was more than tenfold greater in the case of a hypertrophic shallow lake than predicted from the model [14]. APP can exhibit even mass production in hypertrophic lakes, such as in Lake Trummen (Sweden), in a pond near Prosigk (Germany) and in the shallow turbid soda pans of the Carpathian Basin, where APP constituted 90–100% of the total phytoplankton biomass [15–19]. These exceptions show that the importance of APP has been overlooked in numerous productive waters as stated by Carrick and Schelske [14]. Many hypertrophic shallow lakes can be characterized by APP dominance, which contradicts the model of Bell and Kalff [13] and suggest the influence of another factor. Up to now, the cause of these deviations is unknown, but shallowness (<2 m) seems to be a common feature of these systems. As a result of system morphometry and wind-induced mixing, shallow waters can be characterized by high inorganic turbidity. Soda pans of the Carpathian Basin, for example, could have extremely high concentration (>10000 mg l^-1^) of suspended solids (TSS) in the water column [20, 21]. In this paper, our aim was to test whether the relationship between APP abundance/contribution and phytoplankton biomass (chlorophyll *a*) also applies for shallow turbid lakes, or inorganic turbidity can play a complementary role in determining the size distribution of phytoplankton communities, thus explaining the observed deviations from the model of Bell and Kalff [13].

## Materials and methods

### Study sites and sampling

Lake Balaton (Hungary) is the largest shallow lake in Central Europe with a surface area of 596 km^2^ and an average depth of 3.3 m. The lake has relatively high electric conductivity (EC; 730–1000 μS cm^-1^), slightly alkaline water (pH: 8–9) and moderately high inorganic turbidity [22]. Lake Fertő/Neusiedlersee is a wind-exposed, extremely shallow (~ 1 m) steppe lake (EC between 2 and 2.6 mS cm^-1^, pH between 8 and 9) with high inorganic turbidity located at the Austrian/Hungarian border. The total surface area of the lake is 309 km^2^, of which about 55% covered by reed [23]. Turbid soda pans (Unterer Strinkersee, Böddi-szék pan, Fehér-szék pan, Kelemen-szék pan and Zab-szék pan) are intermittent alkaline water bodies (EC between 1 and 24 mS cm^-1^, pH between 8 and 10) with maximum water depth of about 0.5 m, which frequently dry out entirely by the end of the summer [20]. Due to their high inorganic turbidity and high CDOM concentration [22], they have extremely low Secchi-disk transparency (Table 1).

**Table 1:** List of investigated lakes, sampling strategy, selected physical and chemical variables. Abbreviations: H–Hungary, A–Austria, B–biweekly, M–monthly.

Lake	Country	Coordinates	Sampling period	Surface	Mean depth	Electric conductivity		pH		Secchi-disk transparency		Zmix/Zeu	
			and			range	mean	range	mean	range	mean	range	mean
			frequency	(ha)	(cm)	(μS cm^-1^)				(cm)			
Lake Balaton Eastern basin	H	N46°58.267'E18°4.921'	2008–2009, B	22800	370	700–998	813	8.0–9.1	8.57	38–145	75	0.4–2.1	0.9
Lake Balaton Western basin	H	N46°43.652'E17°16.520'	2008–2009, B	3800	230	727–848	747	8.1–9.1	8.56	22–133	54	0.6–3.2	1.3
Lake Fertő/Neusiedlersee	H/A	N47°46.228'E16°43.298'	2008–2009, B	13800^a^	100	1990–2600	2260	8.3–9.3	8.79	3–60	28	0.2–5.8	1.7
Unterer Strinkersee	A	N47°47.762'E16°47.160'	2008–2009, B	36	34	2830–6620	4469	8.7–9.4	9.04	2–47	18	0.5–13	1.6
Böddi-szék pan	H	N46°46.061'E19°8.726'	2013, M	198	12	3190–24200	8827	9.1–10	9.53	2–13	6.3	0.5–2.5	1
Fehér-szék pan	H	N46°48.448'E19°11.221'	2001, 2013, M	10	25	949–9500	4114	8.3–9.7	9.01	1–27	8.0	0.6–6	2.3
Kelemen-szék pan	H	N46°47.542'E19°10.647'	2001, 2013, M	190	23	1475–16900	5783	8.7–10.7	9.35	0.5–8	3.4	1.3–15	4.2
Zab-szék pan	H	N46°50.190'E19°10.283'	2001, 2013, M	182	20	2070–21200	7532	9.2–10.2	9.65	0.5–7	3.0	0.9–7.5	3.2

^a^open water

Water samples were taken from Lake Balaton, from Lake Fertő/Neusiedlersee and from turbid soda pans in Hungary/Austria (Unterer Strinkersee; Böddi-szék pan, Fehér-szék pan, Kelemen-szék pan and Zab-szék pan). Permissions for sampling were obtained from the Balaton Uplands National Park, the Fertő-Hanság National Park and the Kiskunság National Park. Details of sampling period and frequency are described in Table 1.Secchi-disk transparency, EC and pH were measured on the field. Light attenuation within the water column was measured with a LI-COR quantum sensor (2π). The Zmix/Zeu ratio was calculated on the basis of the depth of the entire water column (Zmix) and the depth of the euphotic zone (Zeu), which was calculated from the measured light attenuation (4.6/Kd) according to Kirk [24]

### Measurements

Freshly collected samples were immediately transported into the laboratory. Total suspended solids (TSS) content was determined gravimetrically after sample filtration on 0.4 pore size cellulose acetate filters [25]. Particulate organic carbon (POC) was calculated as the difference between total organic carbon (TOC) and dissolved organic carbon (DOC) concentration. The concentration of TOC and DOC were measured using Elementar High TOC analyser according to V.-Balogh et al. [22, 26]. Particulate organic matter was estimated by assuming a 1:2 ratio between POC and the total dry mass of the organic matter [26]. Subtracting this value from TSS concentration yielded organic matter free suspended solid (TSS-Org) concentration.

Water samples of 10–1000 ml were concentrated on glass fiber filters (Macherey-Nagel; GF-5; nominal pore size is 0.4 μm) and chlorophyll *a* concentration was determined spectrophotometrically after hot methanol extraction using the absorption coefficients determined by Wellburn [27]. The nano- and microplankton samples were fixed by Lugol-solution, their abundance and composition was determined with an inverted microscope [28]. Settled volume varied between 2 and 10 ml depending on nanoplankton abundance and highly turbid waters were diluted two- or fivefold to avoid the masking effect of the inorganic particles. The abundance and composition of APP was determined in fresh, unpreserved samples according to MacIsaac and Stockner [29]. Briefly, the samples were concentrated on 0.4 μm pore size black cellulose-acetate filters (Macherey-Nagel), the filters were embedded into 50% glycerol and the slides were examined with a Nikon Optiphot 2 epifluorescence microscope at 1000 x magnification, using blue-violet (BV-2A) and green (G-2A) excitation light to detect APP cells according to MacIsaac and Stockner [29] At least 20 fields (400 cells) were photographed with a Spot RT colour camera and picoalgae were counted on these pictures to avoid fluorescence fading. The total biovolume of the pico- nano- and microplankton was calculated on the basis of cell volume and abundance values. For picoalgal biomass estimation, the cell diameter of picocyanobacteria and picoeukaryotes was assumed to be 1.0 and 1.5 μm, respectively. The biomass (wet weight) of the different size classes was estimated from the total biovolume of the fractions assuming a specific gravity of 1.0.

### Statistical analysis

In order to compare the present data set to the findings of Bell and Kalff [13], a linear model was used to describe the relationship between phytoplankton biomass and APP abundance/contribution. Chlorophyll *a* was used as a proxy of phytoplankton biomass according to Bell and Kalff [13]. To test the dependence of fitted parameters on inorganic turbidity, the data set was analysed by Model-based recursive partitioning (MOB) [30]. MOB fits a model tree using the following procedure:

1. Fit a linear model between log-transformed chlorophyll *a* concentration and log-transformed abundance/contribution of APP for all observations.
2. Assess the stability of the model parameters with respect to the partitioning variable (here inorganic turbidity) and search for the locally optimal split. If Bonferroni-corrected p-value of the optimal split is smaller than the significance level (α = 0.05), data are divided into two parts, otherwise stop.
3. Re-fit the model in both parts, and repeat from step 2.

To compare parameters of the fitted lines to parameters published by Bell and Kalff [13], 95% confidence intervals were calculated, analysing all data, and the groups created by MOB separately. Assumptions of the linear regression models were checked by drawing diagnostic graphs (Supporting information 2).

Since model-based recursive partitioning does not take the possibility of a non-linear relationship into account and cannot evaluate the relationship between more than two variables (turbidity, phytoplankton biomass measured as chlorophyll *a* and APP contribution), for further exploration of contribution of APP and the effect of inorganic turbidity, conditional inference-based regression tree was fitted to the data. Regression trees are non-parametric statistical methods that can handle nonlinear relationships, and the results are easy to interpret and indicate the variable that significantly discriminates between classes [31]. The selected algorithm offers unbiased variable selection and a statistically sound stopping rule [32], which eliminates the variable selection bias and problems of under- and over-fitting. All statistical analysis were done in R 3.1.1. [33] using party package.

## Results

There were large differences between the studied lakes in terms of their inorganic turbidity. Organic matter free suspended solid concentration ranged between 4 and 49 mg l^-1^ in Lake Balaton and between 7 and 236 mg l^-1^ in Lake Fertő/Neusiedlersee (Table 1). The other water bodies were extremely shallow (<40 cm mean depth) which resulted in much higher maximum TSS-Org concentrations (>2000 mg l^-1^). Underwater light climate of the studied lakes was affected significantly by high inorganic turbidity, particularly in turbid soda pans with low Secchi-disk transparency (Table 1). As a result of their extreme shallowness, however, Zmix/Zeu ratio was only occasionally higher than that of the ‘deeper lakes’ (Table 1). Zmix/Zeu ratio ranged between 0.4 and 3.2 in in Lake Balaton and between 0.2 and 5.8 in Lake Fertő/Neusiedlersee. In the turbid soda pans, Zmix/Zeu ratio was between 0.5 and 15 (Table 1).

Chlorophyll *a* concentration showed high variability in a similar way to inorganic turbidity, ranging between 1 and 460 μg l^-1^ for all of the studied lakes (Table 2). The Eastern basin of Lake Balaton had a mesotrophic character, while the Western basin of the lake and Lake Fertő/Neusiedlersee was eutrophic (OECD fixed boundary system). Unterer Strinkersee could be characterized as a meso-eutrophic water body, while the rest of the sampling sites had a hypertrophic character with maximum chlorophyll *a* values between 80 and 460 μg l^-1^ (Table 2). APP abundance varied between 0 and 100 million cells ml^-1^ in the studied shallow lakes (Table 2). The lowest abundances were detected in Lake Balaton with an average of 0.2 x 10^6^ cells ml^-1^ in the Eastern basin and 0.3 x 10^6^ cells ml^-1^ in the Western basin. Higher APP abundances were found in Lake Fertő/Neusiedlersee (average: 0.5 x 10^6^cells ml^-1^) and Unterer Strinkersee (average: 0.5 x 10^6^ cells ml^-1^). The hypertrophic waters were extremely abundant in APP (Table 2). In the case of Böddi-szék pan and Kelemen-szék pan, the average APP abundance was 3 x 10^6^ cells ml^-1^ and 4 x 10^6^ cells ml^-1^, respectively. Higher average abundances were observed in Fehér-szék pan (14 x 10^6^ cells ml^-1^) and Zab-szék pan (21 x 10^6^ cells ml^-1^). In the latter, the maximum APP abundance exceeded 100 million cells ml^-1^. According to the microscopic images, APP composed of free living cells in the studied shallow lakes. Inorganic suspended particles were in the size range of APP cells or were somewhat smaller (their diameter ranged between c.a. 0.3–4 μm).

**Table 2:** Inorganic turbidity, phytoplankton biomass (wet weight) and composition of the studied lakes. Abbreviations: TSS-Org–Organic matter free suspended solid concentration, APP–autotrophic picoplankton.

Lake	TSS-Org		Chlorophyll *a*		Phytoplankton biomass		APP abundance		APP biomass		APP contribution	
	(mg l^-1^)		(μg l^-1^)		(μg l^-1^)		(10^6^ cells ml^-1^)		(μg l^-1^)		(%)	
	range	mean	range	mean	range	mean	range	mean	range	mean	range	mean
Lake Balaton Eastern basin	4–22	11	2.0–13	6	177–3460	1241	0.06–0.53	0.23	30–293	138	2–68	18
Lake Balaton Western basin	4–49	19	2.3–37	15	187–9508	2756	0.02–0.84	0.28	26–523	190	1–60	18
Lake Fertő/Neusiedlersee	7–236	46	3.5–37	12	293–2171	967	0.09–1.47	0.51	49–769	265	5–69	26
Unterer Strinkersee	9–836	136	0.9–27	7	85–2725	699	0.01–1.29	0.50	5–698	276	3–100	37
Böddi-szék pan	153–1062	506	4.3–164	48	74–13728	2214	0.04–23.1	3.12	74–13728	2090	45–100	86
Fehér-szék pan	9–2252	637	1.7–400	92	8–47918	12015	0.00–64.6	14.24	0–47889	11830	0–100	61
Kelemen-szék pan	313–2160	928	2.6–78	24	53–8883	3402	0.03–13.6	4.11	53–7083	3147	80–100	95
Zab-szék pan	368–2191	1007	1.7–456	121	159–127714	26078	0.08–103	20.71	159–127714	25929	92–100	99

The contribution of APP to total phytoplankton biomass ranged between 1 and 68% in Lake Balaton and between 5 and 70% in Lake Fertő/Neusiedlersee In the extremely shallow waters, exclusive APP dominance was a common phenomenon reaching 100% contribution occasionally (Table 2).

**Fig 1:**
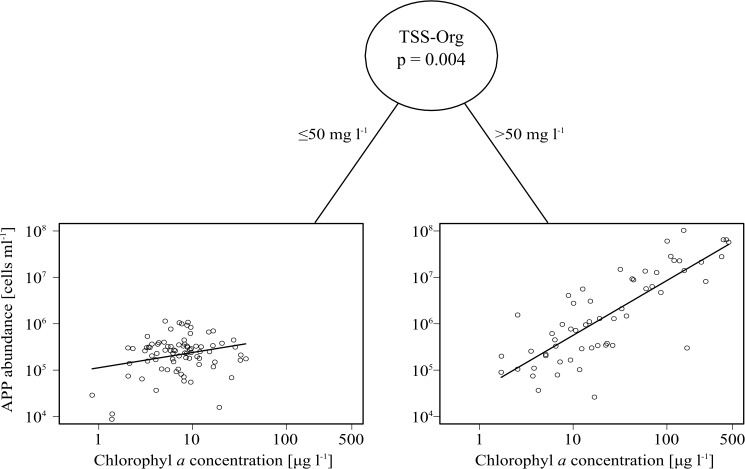
Model-based recursive partitioning of the relationship between chlorophyll *a* concentration and APP abundance using organic matter free suspended solid concentration (TSS-Org) as partitioning variable. The analysis revealed that the parameters of the fitted linear relationship is significantly different for below and above 50 mg l^-1^ sites. Regression lines for the corresponding equations are indicated.

There was a significant positive relationship between APP abundance and chlorophyll *a* concentration regarding all of the studied lakes (Table 3). However, this relationship significantly varied as a function of inorganic turbidity. As shown by the result of the MOB analysis with a locally optimal split in the relationship, below 50 mg l^-1^ TSS-Org concentration, the slope of the regression line was gentler than in more turbid waters (Table 3, Fig 1). The slope and intercept of both regression models were different from the one determined by Bell and Kalff [13]. Our finding on the effect of turbidity dividing the relationship into two separate sections demonstrates that a simple linear model such as the one of Bell and Kalff [13] cannot be applied for shallow turbid lakes.

**Table 3:** Parameters of the lines fitted to log_10_[APP abundance (cells ml^-1^)] vs. log_10_[chlorophyll *a* concentration (μg l^-1^)] relationships.

Ecosystem type	Data source	Intercept			Slope		
		estimate	lower	upper	estimate	lower	upper
			bound of 95% confidence interval			bound of 95% confidence interval	
Freshwaters	Bell and Kalff (2001)	4.16			0.74	0.52	0.96
Shallow lakes	present study	4.50	4.31	4.69	1.11	0.95	1.27
Shallow lakes with TSS-Org≤50 mg l^-1^	present study	5.05	4.80	5.30	0.33	0.05	0.60
Shallow lakes with TSS-Org>50 mg l^-1^	present study	4.57	4.23	4.91	1.18	0.96	1.41

**Fig 2:**
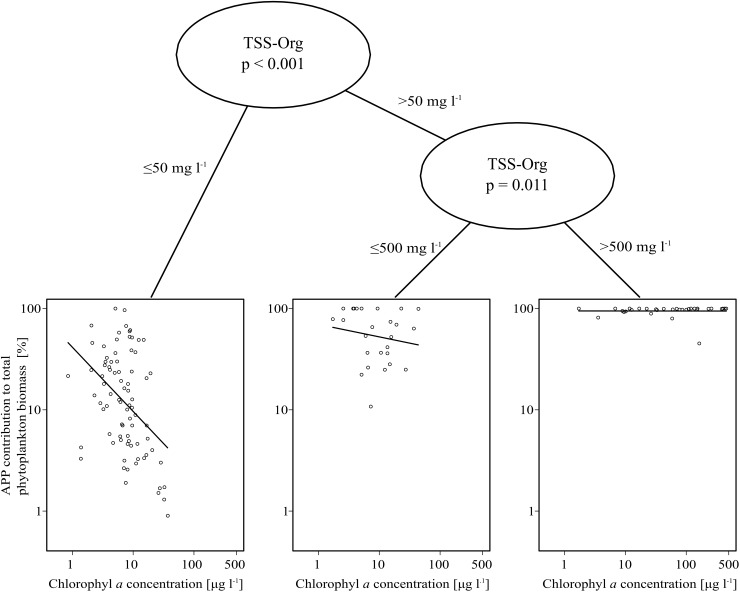
Model-based recursive partitioning of the relationship between chlorophyll a concentration and APP contribution to total phytoplankton biomass using organic matter free suspended solid concentration (TSS-Org) as partitioning variable. The analysis resulted in three groups of sites with significantly different parameters of the fitted line: below 50 mg l^-1^ TSS-Org, between 50 and 500 mg l^-1^ TSS-Org and above 500 mg l^-1^ TSS-Org. Regression lines for the corresponding equations are indicated.

In contrast with the negative relationship described by Bell and Kalff [13], our data set showed a weak (but significant) positive relationship between APP contribution and chlorophyll *a* concentration regarding all of the studied lakes (Table 4). However, on the basis of the MOB analysis, the dataset can be divided into three partitions. Samples with TSS-Org under 50 mg l^-1^ strongly separated from those with a higher TSS-Org (p < 0.001), and there was another significant split (p = 0.011) between samples with 50–500 mg l^-1^ and above 500 mg l^-1^ TSS-Org. When only moderately turbid (<50 mg l^-1^ TSS-Org) conditions were taken into account, the analysis showed a significant strong negative relationship. The obtained regression line had higher intercept and lower slope than the freshwater regression line of Bell and Kalff [13]. Above a TSS-Org concentration of 50 mg l^-1^ there was only a very weak and not significant negative relationship (Table 4, Fig 2.). Under extremely turbid conditions (>500 mg l^-1^ TSS-Org), APP contribution was around 100% regardless of chlorophyll *a* concentration: the slope of the obtained regression line did not differ significantly from 0 (Table 4, Fig 2). These results also confirmed the need for a method such as a regression tree analysis that can handle nonlinear relationships.

**Table 4:** Parameters of the lines fitted to log_10_[APP contribution (%)] vs. log_10_[chlorophyll *a* concentration (μg l^-1^)] relationships. Regression analysis showed no significant relationship above 50 mg l^-1^ TSS-Org (p > 0.05).

Ecosystem type	Data source	Intercept			Slope		
		estimate	lower	upper	estimate	lower	upper
			bound of 95% confidence interval			bound of 95% confidence interval	
Freshwaters	Bell and Kalff (2001)	1.56			-0.53	-0.43	-0.64
Shallow lakes	present study	1.14	0.94	1.33	0.25	0.08	0.41
Shallow lakes with TSS-Org≤50 mg l^-1^	present study	1.62	1.33	1.90	-0.63	-0.95	-0.32
Shallow lakes with TSS-Org between 50 and 500 mg l^-1^	present study	1.84	1.53	2.15	-0.12	-0.43	0.18
Shallow lakes with TSS-Org>500 mg l^-1^	present study	1.98	1.91	2.04	-0.0004	-0.04	0.04

**Fig 3:**
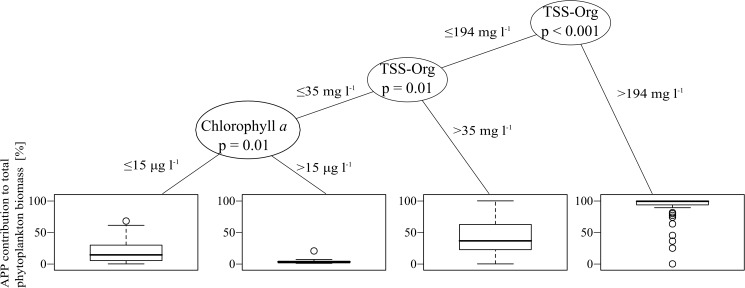
Regression tree model on the effect of trophic status (chlorophyll *a* concentration) and inorganic turbidity (TSS-Org) on APP contribution (%) in shallow lakes.

Regression tree analysis clearly demonstrated that turbidity was the key factor in determining APP contribution in shallow turbid lakes (Fig 3). The analysis yielded four distinct categories in terms of TSS-Org and chlorophyll *a*. High inorganic turbidity (>194 mg l^-1^ TSS-Org) regularly caused high APP contribution (with a mean of 90%) regardless of the chlorophyll *a* concentration. At moderate turbidity (35–194 mg l^-1^), APP contribution was in general lower and showed higher variability with a mean of 40%, also independently of chlorophyll *a* concentration. On the other hand, samples with TSS-Org under 35 mg l^-1^ were even lower and could be divided into two classes according to their chlorophyll *a* concentration: at higher values (>15 μg l^-1^), APP contribution was always low (with a mean of 5%), while at lower values APP contribution showed higher variability with a mean of 20% (Fig 3).

## Discussion

In contrast with the generally accepted view [7–10] that phytoplankton in highly productive waters is dominated by larger-sized (> 3 μm) species, APP dominance was described in shallow, turbid lakes regardless of phytoplankton biomass. Our results clearly demonstrated that a simple linear regression model between phytoplankton biomass and APP abundance/contribution cannot describe the relationship in this type of water bodies. Thus, the generally accepted model of Bell and Kalff [13], which was successfully applied in marine and certain freshwater environments, is not suitable for shallow turbid lakes. Regarding APP abundance, we found higher values than in other freshwater lakes with the same phytoplankton biomass [13], particularly above 50 mg l^-1^ TSS-Org concentration. As for the contribution of APP to total phytoplankton biomass, turbidity has a decisive role influencing size distribution within the phytoplankton of shallow lakes. According to the MOB analysis, the relative importance of APP decreased with increasing chlorophyll *a* in moderately turbid (<50 mg l^-1^ TSS-Org) waters, with a tendency similar to those observed in other continental waters [8–10], but with higher APP contribution (Table 4). In more turbid waters, however, significant relationship was not found. Thus, our results have indicated an ecological switching point (50 mg l^-1^), above which the generally accepted rule suggested by the model of Bell and Kalff [13] (decreasing APP contribution with increasing chlorophyll *a*) does not apply for shallow turbid aquatic environments. On the basis of TSS-Org and chlorophyll *a*, the non-parametric regression tree analysis yielded four categories with characteristic APP contribution. These categories form a series of conditions with low TSS-Org, high chlorophyll *a* (> 15 μg l^-1^) and very low APP contribution at one end and with high TSS-Org and constantly high APP contribution (90%) at the other.

**Fig 4:**
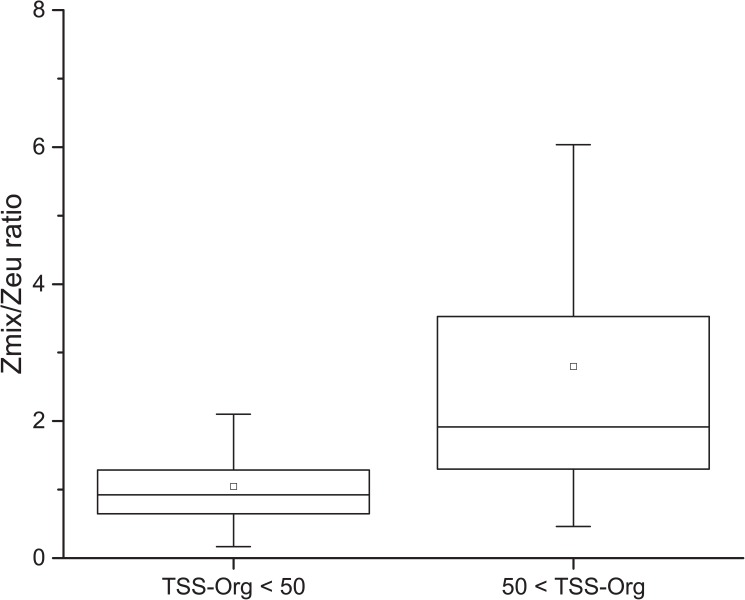
Boxplot of mixing to euphotic depth (Zmix/Zeu) ratio measured in the studied shallow lakes below and above the ecological switching point of 50 mg l^-1^ TSS-Org. Whiskers extend to 5 ^th^ and 95^th^ percentiles, boxes represent lower and upper quartiles, horizontal lines are medians, mean values are marked with squares.

The occurrence and dynamics of APP in aquatic ecosystems is influenced by both bottom-up (e.g. nutrient supply, PAR, UV-B, genome streamlining) and top-down control (sinking, biophagy and grazing) [2,34]. Inorganic turbidity strongly restricts the availability of PAR in shallow lakes [24,35] and may have also a significant effect on grazing. Both mechanisms could promote APP success. Availability of PAR can be characterized with the mixing to euphotic depth (Zmix/Zeu) ratio, reaching usually higher values in more turbid waters. Extremely high Zmix/Zeu ratio has a strong negative influence on productivity due to losses in the aphotic zone as a result of dark metabolism or increased respiration [36]. Shallow turbid soda pans, however, are highly productive waters despite their extremely high inorganic turbidity. Pálffy et al. [19] found a winter APP biomass peak with a chlorophyll *a* concentration of ca. 1000 μg l^-1^ and a daily primary production of 180 mg C m^-2^ d^-1^ in a shallow, ice-covered turbid soda pan. According to Talling [37] and Grobbelaar [38] natural phytoplankton populations are not capable of net photosynthesis when the Zmix/Zeu ratio exceeds a value of 5 and 5.7, respectively. Zmix/Zeu ratio of the studied lakes were usually below these critical values (Table 1), however, Zmix/Zeu ratio increased with increasing turbidity. According to the switching point determined by the MOB analysis, considerably lower Zmix/Zeu ratio was observed in case of samples with TSS-Org under 50 mg l^-1^ (1 on average), while at higher values (2.8 on average) as shown in Fig 4. This gradual shift suggest increasing light limitation along a turbidity range in the studied shallow lakes.

Low light conditions generally mean competitive advantage for autotrophic picoplankton over larger-sized phytoplankton. Cell size is considered a master trait influencing several species characteristics [39–41], including light-growth responses, i.e. low light conditions generally mean competitive advantage for autotrophic picoplankton over larger-sized phytoplankton. Owing to the reduced chromophore self-shading of smaller cells [2,42], light harvesting at low photon flux densities generally works more efficiently in APP, which eventually leads to a higher specific growth rate as compared to larger-celled components of the phytoplankton [42]. A lowlight-adapted APP strain can have a light-saturation parameter of as low as 3 μmol m^-2^ s^-1^ [43]. Based on fractionated photosynthesis measurements, APP was better acclimated to low light than larger-sized phytoplankton, which was confirmed by their higher light utilization parameter [44,45]. This low light acclimation of APP was particularly apparent in a shallow turbid alkaline pan [19]. Adaptation of APP cells to light-limited environment in deep lakes was also supported by the increase of cellular chlorophyll a content [46] and cellular photosynthetic efficiency [47] under low-light intensities. This is in agreement with the finding that APP contribution to total primary production often increases with depth. Such a tendency was found in a meromictic lake, where the contribution of APP to primary production varied between 44 and 97% in deeper layers with particularly low (<10 μmol m^-2^ s^-1^) light intensity [48]. The spatial distribution of their abundance generally shows a similar pattern with maxima often occurring near the bottom of the euphotic zone (1% of surface PAR) in deep waters [49]. APP dominance was also found in the deep chlorophyll maximum of the holomictic Lake Stechlin [50], although the authors explained the phenomenon with the peculiar pigment composition of picocyanobacteria, while their small size was assumed to confer competitive advantage through more efficient nutrient uptake. The ability to saturate photosynthesis and growth rate at very low irradiances was also confirmed for marine APP [51,52], contributing more to primary productivity at lower than at higher light intensities, further supported by studies that found their maximum abundance to be in lowlight environments [53,54].

In addition to the regulating effect of light, grazing is considered to be a similarly important processes influencing phytoplankton size structure [2,34]. Protozoa, namely heterotrophic flagellates and ciliates, and small metazoa such as rotifers have been identified as the primary picoplankton grazers. However, in some cases, APP dynamics is mainly controlled by daphnids [55–57]. What makes APP a separate group within the phytoplankton is the grazer-prey dynamics, which is considerably different as compared to larger cells. According to the size differential grazing concept, due to the fact that the generation times of APP grazers (protozoa) are similar to those of their prey, APP biomass is more tightly controlled by grazing than larger phytoplankton, whose consumers have relatively longer generation times [58,59]. The above mechanism prevents APP dominance in mesotrophic and eutrophic environments, when APP stay under control of their protist grazers, while larger phytoplankton can produce larger biomass [39]. As a result, pPicoplankton peaks could occur under these conditions only when APP grazing is reduced [39]. Thus, the reduction of grazing pressure is another possible explanation for APP dominance in shallow turbid waters.

This assumption is also supported by several authors, who have demonstrated the decline of zooplankton feeding with increasing turbidity in either field or laboratory studies. In Lake Balaton, for example, the clearance rate of *Daphnia galeata* decreased at TSS concentrations higher than 25 mg l^-1^ [60]. Feeding experiments also showed that clay (at 50 mg l^-1^ concentration) can significantly decrease the phytoplankton ingestion rates of five cladoceran species [61,62]. The filtration rate of the rotifer *Brachionus calyciflorus* and the growth rate of the ciliate *Strobilidium gyrans* was also supressed by suspended particles at a concentration of 50 mg l^-1^ or 100 mg l^-1^, respectively [63,64]. Pfandl and Boenigk [65] studied the effect of suspended particles (up to 10 mg l^-1^) on colourless chrysomonad flagellates. It has been found, that small suspended particles in the size range of ingestible bacteria interfere with the feeding process of flagellates and cause lower clearance rates [65,66].

**Fig 5:**
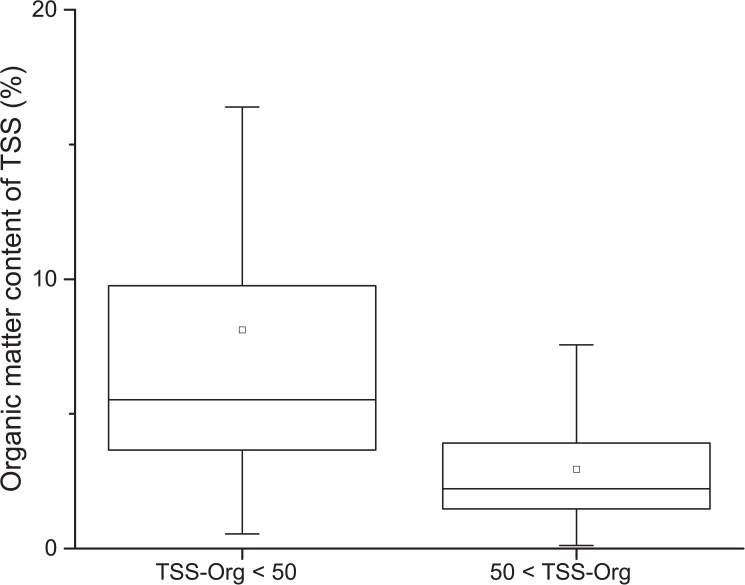
Boxplot of organic matter content of TSS measured in the studied shallow lakes below and above the ecological switching point of 50 mg l^-1^ TSS-Org. Whiskers extend to 5 ^th^ and 95^th^ percentiles, boxes represent lower and upper quartiles, horizontal lines are medians, mean values are marked with squares.

Decreasing grazing pressure as a function of increasing turbidity is usually explained by the relative decrease of food to inorganic particles. According to our results, the organic matter content of TSS decreases with the increase of TSS concentration (Fig 5). Consistent with the switching point determined by the MOB analysis, the organic matter content of TSS was considerably higher (8% on average) in case of samples with TSS-Org under 50 mg l^-1^ than at above (3% on average). High abundances of particles in the size range of potential food particles can lead to reduced population growth rates or even dying back of zooplankton. When suspended sediment concentration exceeds the threshold below which zooplankton species can effectively filter material, the available food is diluted and the organisms may starve even at high food abundance [66].

## Conclusions

Our results clearly described the unusual behaviour of the APP community in extremely shallow lakes and pans. Empirical evidence was found for a turbidity-related ecological switching point (50 mg l^-1^), above which APP contribution to phytoplankton biomass showed an increasing trend with increasing inorganic turbidity. The negative relationship observed by Bell and Kalff [13] between APP contribution and total phytoplankton biomass can only be observed below that critical turbidity value. We hypothesize that high turbidity indirectly affects APP either by diminishing the underwater light intensity or by reducing grazing pressure. Specific laboratory experiments (e.g. grazing studies as a function of inorganic turbidity) will be necessary to test this assumption.

## Supporting information

S1 Data Supplementary material on measured parameters. Organic matter free suspended solid concentration (TSS-Org), chlorophyll *a* concentration and autotrophic picoplankton (APP) abundance, biomass and contribution to total phytoplankton biomass in the studied lakes.
(PDF)

S1 Fig Diagnostic plots of linear regression models. Assumptions of linear regressions (i.e homoscedasticity and normality of residuals) and lack of influential points were checked by drawing the standard diagnostic plots of R.
(PDF)
