# Supplementary material for: Unusual behaviour of phototrophic picoplankton in turbid waters
Source: PLoS One. 2017 Mar 27;12(3):e0174316. doi: 10.1371/journal.pone.0174316 (PMC5367718; doi:10.1371/journal.pone.0174316)
Supplement: S1 Data — Organic matter free suspended solid concentration (TSS-Org), chlorophyll a concentration and autotrophic picoplankton (APP) abundance, biomass and contribution to total phytoplankton biomass in the studied lakes. (PDF) [file pone.0174316.s001.pdf]

Supplementary material on measured parameters (Somogyi et al.)

Lake	Coordinates	Date	TSS-Org (mg/L)	Chlorophyll a (ug/l)	APP abundance (cells/ml)	APP biomass (µg/l)	APP contribution (%)
Zab-szék pan	N46°50.190'; E19°10.283'	17/04/2013	1493	6.80	78597	159	100
Zab-szék pan	N46°50.190'; E19°10.283'	30/05/2013	786	11.73	102176	207	100
Zab-szék pan	N46°50.190'; E19°10.283'	18/06/2013	640	1.71	89825	160	100
Zab-szék pan	N46°50.190'; E19°10.283'	24/07/2013	368	3.83	110036	176	100
Zab-szék pan	N46°50.190'; E19°10.283'	15/08/2013	438	44.05	8829063	5811	99
Zab-szék pan	N46°50.190'; E19°10.283'	24/09/2013	2191	153.24	14068863	18449	100
Zab-szék pan	N46°50.190'; E19°10.283'	17/10/2013	1991	259.82	8160989	14998	100
Zab-szék pan	N46°50.190'; E19°10.283'	19/11/2013	1638	430.18	64449540	127714	100
Zab-szék pan	N46°50.190'; E19°10.283'	09/12/2013	897	455.74	57288480	114633	100
Zab-szék pan	N46°50.190'; E19°10.283'	06/04/2001	1123	86.30	4718000	10253	97
Zab-szék pan	N46°50.190'; E19°10.283'	26/05/2001	762	31.86	14868000	18788	98
Zab-szék pan	N46°50.190'; E19°10.283'	16/06/2001	517	110.00	28428000	18742	99
Zab-szék pan	N46°50.190'; E19°10.283'	19/07/2001	1055	59.94	5679450	3895	98
Zab-szék pan	N46°50.190'; E19°10.283'	14/08/2001	601	150.98	103079200	53601	98
Zab-szék pan	N46°50.190'; E19°10.283'	16/10/2001	611	9.45	768000	1352	92

Supplementary material on measured parameters (Somogyi et al.)

Lake	Coordinates	Date	TSS-Org (mg/L)	Chlorophyll a (ug/l)	APP abundance (cells/ml)	APP biomass (µg/l)	APP contribution (%)
Kelemen-szék pan	N46°47.542'; E19°10.647'	17/04/2013	2151	16.88	26199	53	100
Kelemen-szék pan	N46°47.542'; E19°10.647'	30/05/2013	431	3.72	74667	151	100
Kelemen-szék pan	N46°47.542'; E19°10.647'	18/06/2013	369	2.56	104796	213	100
Kelemen-szék pan	N46°47.542'; E19°10.647'	24/07/2013	313	9.37	165054	299	100
Kelemen-szék pan	N46°47.542'; E19°10.647'	15/08/2013	580	42.63	9274446	5610	99
Kelemen-szék pan	N46°47.542'; E19°10.647'	06/04/2001	2160	33.00	2111171	5803	97
Kelemen-szék pan	N46°47.542'; E19°10.647'	26/05/2001	933	8.92	4071200	5583	95
Kelemen-szék pan	N46°47.542'; E19°10.647'	16/06/2001	734	10.10	2760000	2760	94
Kelemen-szék pan	N46°47.542'; E19°10.647'	19/07/2001	860	77.75	12711150	6610	98
Kelemen-szék pan	N46°47.542'; E19°10.647'	14/08/2001	701	58.75	13621180	7083	80
Kelemen-szék pan	N46°47.542'; E19°10.647'	16/10/2001	978	3.56	256000	451	81
Böddi-szék pan	N46°46.061'; E19°8.726'	17/04/2013	257	4.26	36679	74	100
Böddi-szék pan	N46°46.061'; E19°8.726'	30/05/2013	532	22.37	345827	702	100
Böddi-szék pan	N46°46.061'; E19°8.726'	18/06/2013	400	23.00	377266	765	100
Böddi-szék pan	N46°46.061'; E19°8.726'	24/07/2013	226	5.11	204352	190	100

Supplementary material on measured parameters (Somogyi et al.)

Lake	Coordinates	Date	TSS-Org (mg/L)	Chlorophyll a (ug/l)	APP abundance (cells/ml)	APP biomass (µg/l)	APP contribution (%)
Böddi-szék pan	N46°46.061'; E19°8.726'	15/08/2013	1062	119.36	23087869	13728	100
Böddi-szék pan	N46°46.061'; E19°8.726'	24/09/2013	589	163.91	299833	595	45
Böddi-szék pan	N46°46.061'; E19°8.726'	19/11/2013	153	15.76	301289	243	52
Böddi-szék pan	N46°46.061'; E19°8.726'	09/12/2013	825	26.40	340587	419	89
Fehér-szék pan	N46°48.448'; E19°11.221'	06/04/2001	776	69.60	6314000	6374	100
Fehér-szék pan	N46°48.448'; E19°11.221'	26/05/2001	557	12.65	5591600	6303	97
Fehér-szék pan	N46°48.448'; E19°11.221'	16/06/2001	381	2.56	1545600	1546	77
Fehér-szék pan	N46°48.448'; E19°11.221'	19/07/2001	323	15.28	3065100	1594	74
Fehér-szék pan	N46°48.448'; E19°11.221'	14/08/2001	573	100.64	60374960	31395	97
Fehér-szék pan	N46°48.448'; E19°11.221'	16/10/2001	131	1.72	200000	352	79
Fehér-szék pan	N46°48.448'; E19°11.221'	28/04/2013	30	2.13	0	0	0
Fehér-szék pan	N46°48.448'; E19°11.221'	30/05/2013	18	3.70	0	0	0
Fehér-szék pan	N46°48.448'; E19°11.221'	18/06/2013	9	2.56	0	0	0
Fehér-szék pan	N46°48.448'; E19°11.221'	24/07/2013	194	3.98	0	0	0
Fehér-szék pan	N46°48.448'; E19°11.221'	15/08/2013	860	13.65	0	0	0

Supplementary material on measured parameters (Somogyi et al.)

Lake	Coordinates	Date	TSS-Org (mg/L)	Chlorophyll a (ug/l)	APP abundance (cells/ml)	APP biomass (µg/l)	APP contribution (%)
Fehér-szék pan	N46°48.448'; E19°11.221'	24/09/2013	2252	400.28	64624200	47889	100
Fehér-szék pan	N46°48.448'; E19°11.221'	17/10/2013	1571	383.40	27875736	45353	99
Fehér-szék pan	N46°48.448'; E19°11.221'	19/11/2013	1137	232.85	21273588	16984	99
Fehér-szék pan	N46°48.448'; E19°11.221'	09/12/2013	747	136.30	22705800	19653	100
Unterer Strinkersee	N47°47.762'; E16°47.160'	14/10/2008	43	5.11	1139878	593	100
Unterer Strinkersee	N47°47.762'; E16°47.160'	27/10/2008	414	13.62	946413	492	36
Unterer Strinkersee	N47°47.762'; E16°47.160'	10/11/2008	500	27.25	1285414	680	25
Unterer Strinkersee	N47°47.762'; E16°47.160'	24/11/2008	37	7.67	992651	516	67
Unterer Strinkersee	N47°47.762'; E16°47.160'	09/12/2008	97	13.62	945784	566	42
Unterer Strinkersee	N47°47.762'; E16°47.160'	22/12/2008	168	10.65	717853	390	37
Unterer Strinkersee	N47°47.762'; E16°47.160'	12/01/2009	39	7.23	1051235	698	96
Unterer Strinkersee	N47°47.762'; E16°47.160'	02/03/2009	23	5.11	268976	186	36
Unterer Strinkersee	N47°47.762'; E16°47.160'	09/03/2009	836		434903	240	
Unterer Strinkersee	N47°47.762'; E16°47.160'	14/04/2009	18	0.85	28819	18	21.58
Unterer Strinkersee	N47°47.762'; E16°47.160'	27/04/2009	28	2.89	64624	34	11.63

Supplementary material on measured parameters (Somogyi et al.)

Lake	Coordinates	Date	TSS-Org (mg/L)	Chlorophyll a (ug/l)	APP abundance (cells/ml)	APP biomass (µg/l)	APP contribution (%)
Unterer Strinkersee	N47°47.762'; E16°47.160'	18/05/2009	9	1.38	8733	5	3.29
Unterer Strinkersee	N47°47.762'; E16°47.160'	02/06/2009	16	2.13	139728	97	45.77
Unterer Strinkersee	N47°47.762'; E16°47.160'	16/06/2009	40	4.05	168110	107	26.41
Unterer Strinkersee	N47°47.762'; E16°47.160'	29/06/2009	9	1.39	11353	6	4.25
Unterer Strinkersee	N47°47.762'; E16°47.160'	14/07/2009	33	4.09	36679	24	5.76
Unterer Strinkersee	N47°47.762'; E16°47.160'	28/07/2009	91	5.11	218325	114	22.22
Unterer Strinkersee	N47°47.762'; E16°47.160'	12/08/2009	54	7.67	967616	503	65.60
Unterer Strinkersee	N47°47.762'; E16°47.160'	24/08/2009	159	6.39	449313	234	36.56
Unterer Strinkersee	N47°47.762'; E16°47.160'	07/09/2009	142	7.25	150208	78	10.77
Unterer Strinkersee	N47°47.762'; E16°47.160'	21/09/2009	126	6.53	328361	171	26.15
Unterer Strinkersee	N47°47.762'; E16°47.160'	05/10/2009	120	5.96	614585	320	53.62
Lake Fertő/Neusiedlersee	N47°46.228'; E16°43.298'	22/09/2008	23	5.84	261466	137	23.63
Lake Fertő/Neusiedlersee	N47°46.228'; E16°43.298'	14/10/2008	12	3.51	307453	161	29.80
Lake Fertő/Neusiedlersee	N47°46.228'; E16°43.298'	27/10/2008	50	9.65	240003	126	12.67
Lake Fertő/Neusiedlersee	N47°46.228'; E16°43.298'	10/11/2008	31	16.61	699663	367	20.54

Supplementary material on measured parameters (Somogyi et al.)

Lake	Coordinates	Date	TSS-Org (mg/L)	Chlorophyll a (ug/l)	APP abundance (cells/ml)	APP biomass (µg/l)	APP contribution (%)
Lake Fertő/Neusiedlersee	N47°46.228'; E16°43.298'	24/11/2008	82	12.35	288771	151	24.88
Lake Fertő/Neusiedlersee	N47°46.228'; E16°43.298'	09/12/2008	28	8.95	1075469	564	61.20
Lake Fertő/Neusiedlersee	N47°46.228'; E16°43.298'	22/12/2008	21	14.91	662835	347	49.08
Lake Fertő/Neusiedlersee	N47°46.228'; E16°43.298'	12/01/2009	7	5.86	768504	403	57.88
Lake Fertő/Neusiedlersee	N47°46.228'; E16°43.298'	02/03/2009	13	8.74	927445	486	52.60
Lake Fertő/Neusiedlersee	N47°46.228'; E16°43.298'	09/03/2009	236	37.05	1467144	769	63.46
Lake Fertő/Neusiedlersee	N47°46.228'; E16°43.298'	14/04/2009	62	14.9	1108121	581	28.16
Lake Fertő/Neusiedlersee	N47°46.228'; E16°43.298'	27/04/2009	140	19.17	1292484	678	69.31
Lake Fertő/Neusiedlersee	N47°46.228'; E16°43.298'	18/05/2009	35	8.3	234821	123	15.45
Lake Fertő/Neusiedlersee	N47°46.228'; E16°43.298'	02/06/2009	34	8.51	185334	97	4.92
Lake Fertő/Neusiedlersee	N47°46.228'; E16°43.298'	16/06/2009	40	17.32	149431	78	5.19
Lake Fertő/Neusiedlersee	N47°46.228'; E16°43.298'	29/06/2009	18	10.9	327973	172	8.89
Lake Fertő/Neusiedlersee	N47°46.228'; E16°43.298'	14/07/2009	13	4.26	361934	190	14.31
Lake Fertő/Neusiedlersee	N47°46.228'; E16°43.298'	28/07/2009	95	18.31	338646	178	
Lake Fertő/Neusiedlersee	N47°46.228'; E16°43.298'	12/08/2009	28	8.86	311768	163	8.20

Supplementary material on measured parameters (Somogyi et al.)

Lake	Coordinates	Date	TSS-Org (mg/L)	Chlorophyll a (ug/l)	APP abundance (cells/ml)	APP biomass (µg/l)	APP contribution (%)
Lake Fertő/Neusiedlersee	N47°46.228'; E16°43.298'	24/08/2009	24	11.75	132243	69	4.61
Lake Fertő/Neusiedlersee	N47°46.228'; E16°43.298'	07/09/2009	28	16.69	117896	62	7.00
Lake Fertő/Neusiedlersee	N47°46.228'; E16°43.298'	21/09/2009	21	6.71	92570	49	7.00
Lake Fertő/Neusiedlersee	N47°46.228'; E16°43.298'	05/10/2009	14	9.65	289936	152	7.00
Lake Balaton Eastern basin	N46°58.267'; E18°4.921'	16/01/2008		4.26	212212	293	40.28
Lake Balaton Eastern basin	N46°58.267'; E18°4.921'	12/02/2008	13	3.31	156539	124	18.02
Lake Balaton Eastern basin	N46°58.267'; E18°4.921'	08/04/2008	22	4.97	400321	224	23.18
Lake Balaton Eastern basin	N46°58.267'; E18°4.921'	07/05/2008	11	3.3	534169	287	42.37
Lake Balaton Eastern basin	N46°58.267'; E18°4.921'	03/06/2008	14	4.37	391488	212	29.74
Lake Balaton Eastern basin	N46°58.267'; E18°4.921'	16/06/2008	13	6.18	231599	121	11.95
Lake Balaton Eastern basin	N46°58.267'; E18°4.921'	01/07/2008	15	6.34	270141	142	19.29
Lake Balaton Eastern basin	N46°58.267'; E18°4.921'	07/07/2008	18	6.53	260942	137	7.18
Lake Balaton Eastern basin	N46°58.267'; E18°4.921'	29/07/2008	6	3.69	364166	191	10.89
Lake Balaton Eastern basin	N46°58.267'; E18°4.921'	11/08/2008	16	7.18	202256	106	3.15
Lake Balaton Eastern basin	N46°58.267'; E18°4.921'	26/08/2008	17	11.18	195445	102	2.95

Supplementary material on measured parameters (Somogyi et al.)

Lake	Coordinates	Date	TSS-Org (mg/L)	Chlorophyll a (ug/l)	APP abundance (cells/ml)	APP biomass (µg/l)	APP contribution (%)
Lake Balaton Eastern basin	N46°58.267'; E18°4.921'	09/09/2008	11	9.26	180249	94	4.42
Lake Balaton Eastern basin	N46°58.267'; E18°4.921'	23/09/2008	12	7.56	81508	43	1.90
Lake Balaton Eastern basin	N46°58.267'; E18°4.921'	07/10/2008	14	6.28	151372	79	5.03
Lake Balaton Eastern basin	N46°58.267'; E18°4.921'	29/10/2008	10	7.98	330606	173	10.06
Lake Balaton Eastern basin	N46°58.267'; E18°4.921'	26/11/2008	10	3.69	203188	113	32.62
Lake Balaton Eastern basin	N46°58.267'; E18°4.921'	05/12/2008		5.67	97227	65	15.00
Lake Balaton Eastern basin	N46°58.267'; E18°4.921'	26/01/2009	5	2.06	74405	120	67.99
Lake Balaton Eastern basin	N46°58.267'; E18°4.921'	18/02/2009		13.34	81217	133	22.02
Lake Balaton Eastern basin	N46°58.267'; E18°4.921'	26/03/2009	11	5.39	101652	172	49.48
Lake Balaton Eastern basin	N46°58.267'; E18°4.921'	14/04/2009	5	4.16	229241	169	24.85
Lake Balaton Eastern basin	N46°58.267'; E18°4.921'	13/05/2009	18	3.41	313639	176	27.54
Lake Balaton Eastern basin	N46°58.267'; E18°4.921'	15/06/2009	8	3.24	305218	160	10.13
Lake Balaton Eastern basin	N46°58.267'; E18°4.921'	20/07/2009	16	6.13	173961	91	5.46
Lake Balaton Eastern basin	N46°58.267'; E18°4.921'	17/08/2009	4	2.05	301289	158	24.67
Lake Balaton Eastern basin	N46°58.267'; E18°4.921'	28/09/2009	6	7.04	104796	55	2.65

Supplementary material on measured parameters (Somogyi et al.)

Lake	Coordinates	Date	TSS-Org (mg/L)	Chlorophyll a (ug/l)	APP abundance (cells/ml)	APP biomass (µg/l)	APP contribution (%)
Lake Balaton Eastern basin	N46°58.267'; E18°4.921'	12/10/2009	12	5.88	323121	169	12.56
Lake Balaton Eastern basin	N46°58.267'; E18°4.921'	10/11/2009	5	8.11	58162	30	4.60
Lake Balaton Eastern basin	N46°58.267'; E18°4.921'	07/12/2009	7	4.68	105844	56	4.71
Lake Balaton Western basin	N46°43.652'; E17°16.520'	16/01/2008		3.66	175009	78	22.35
Lake Balaton Western basin	N46°43.652'; E17°16.520'	12/02/2008	4	9.37	276836	243	38.69
Lake Balaton Western basin	N46°43.652'; E17°16.520'	10/03/2008	7	9.48	618296	220	23.88
Lake Balaton Western basin	N46°43.652'; E17°16.520'	08/04/2008	44	8.74	330107	345	59.53
Lake Balaton Western basin	N46°43.652'; E17°16.520'	07/05/2008	21	5.45	323820	177	30.00
Lake Balaton Western basin	N46°43.652'; E17°16.520'	03/06/2008	27	7.16	350019	174	16.31
Lake Balaton Western basin	N46°43.652'; E17°16.520'	16/06/2008	23	8.69	233171	184	11.09
Lake Balaton Western basin	N46°43.652'; E17°16.520'	01/07/2008	17	16.5	337443	122	3.58
Lake Balaton Western basin	N46°43.652'; E17°16.520'	07/07/2008	18	28.54	313340	177	3.01
Lake Balaton Western basin	N46°43.652'; E17°16.520'	29/07/2008	10	15.16	249414	164	3.33
Lake Balaton Western basin	N46°43.652'; E17°16.520'	11/08/2008	49	32.72	211688	131	1.72
Lake Balaton Western basin	N46°43.652'; E17°16.520'	26/08/2008	14	32.38	163482	111	1.30

Supplementary material on measured parameters (Somogyi et al.)

Lake	Coordinates	Date	TSS-Org (mg/L)	Chlorophyll a (ug/l)	APP abundance (cells/ml)	APP biomass (µg/l)	APP contribution (%)
Lake Balaton Western basin	N46°43.652'; E17°16.520'	09/09/2008	18	37.15	175242	86	0.90
Lake Balaton Western basin	N46°43.652'; E17°16.520'	23/09/2008	12	26.08	68641	92	1.51
Lake Balaton Western basin	N46°43.652'; E17°16.520'	07/10/2008	12	8.15	445383	41	5.52
Lake Balaton Western basin	N46°43.652'; E17°16.520'	29/10/2008	31	10.9	0	233	37.06
Lake Balaton Western basin	N46°43.652'; E17°16.520'	26/11/2008	8	11.82	179027	0	0.00
Lake Balaton Western basin	N46°43.652'; E17°16.520'	15/12/2008	44	19.3	15719	179	22.93
Lake Balaton Western basin	N46°43.652'; E17°16.520'	26/01/2009	5	2.31	291100	26	13.88
Lake Balaton Western basin	N46°43.652'; E17°16.520'	19/02/2009			281203	523	49.72
Lake Balaton Western basin	N46°43.652'; E17°16.520'	26/03/2009	6	9.58	837320	394	51.76
Lake Balaton Western basin	N46°43.652'; E17°16.520'	14/04/2009	17	12.35	318318	515	49.01
Lake Balaton Western basin	N46°43.652'; E17°16.520'	13/05/2009	32	8.23	363511	185	18.00
Lake Balaton Western basin	N46°43.652'; E17°16.520'	15/06/2009	4	3.11	260826	191	21.48
Lake Balaton Western basin	N46°43.652'; E17°16.520'	20/07/2009	12	12.01	250200	131	3.26
Lake Balaton Western basin	N46°43.652'; E17°16.520'	10/08/2009		21.78	158358	252	4.59
Lake Balaton Western basin	N46°43.652'; E17°16.520'	31/08/2009	21	27.44	445383	83	1.68

Supplementary material on measured parameters (Somogyi et al.)

Lake	Coordinates	Date	TSS-Org (mg/L)	Chlorophyll a (ug/l)	APP abundance (cells/ml)	APP biomass (µg/l)	APP contribution (%)
Lake Balaton Western basin	N46°43.652'; E17°16.520'	28/09/2009	21	20.62	379886	233	4.00
Lake Balaton Western basin	N46°43.652'; E17°16.520'	12/10/2009	17	9.59	55018	199	10.50
Lake Balaton Western basin	N46°43.652'; E17°16.520'	10/11/2009		8.09	71552	29	2.57
