# Supplementary material for: Unusual behaviour of phototrophic picoplankton in turbid waters
Source: PLoS One. 2017 Mar 27;12(3):e0174316. doi: 10.1371/journal.pone.0174316 (PMC5367718; doi:10.1371/journal.pone.0174316)
Supplement: S1 Fig — Assumptions of linear regressions (i.e homoscedasticity and normality of residuals) and lack of influential points were checked by drawing the standard diagnostic plots of R. (PDF) [file pone.0174316.s002.pdf]

S2 Figs. Diagnostic plots of linear regression models.

Assumptions of linear regressions (i.e homoscedasticity and normality of residuals) and lack of influential points were checked by drawing the standard diagnostic plots of R.

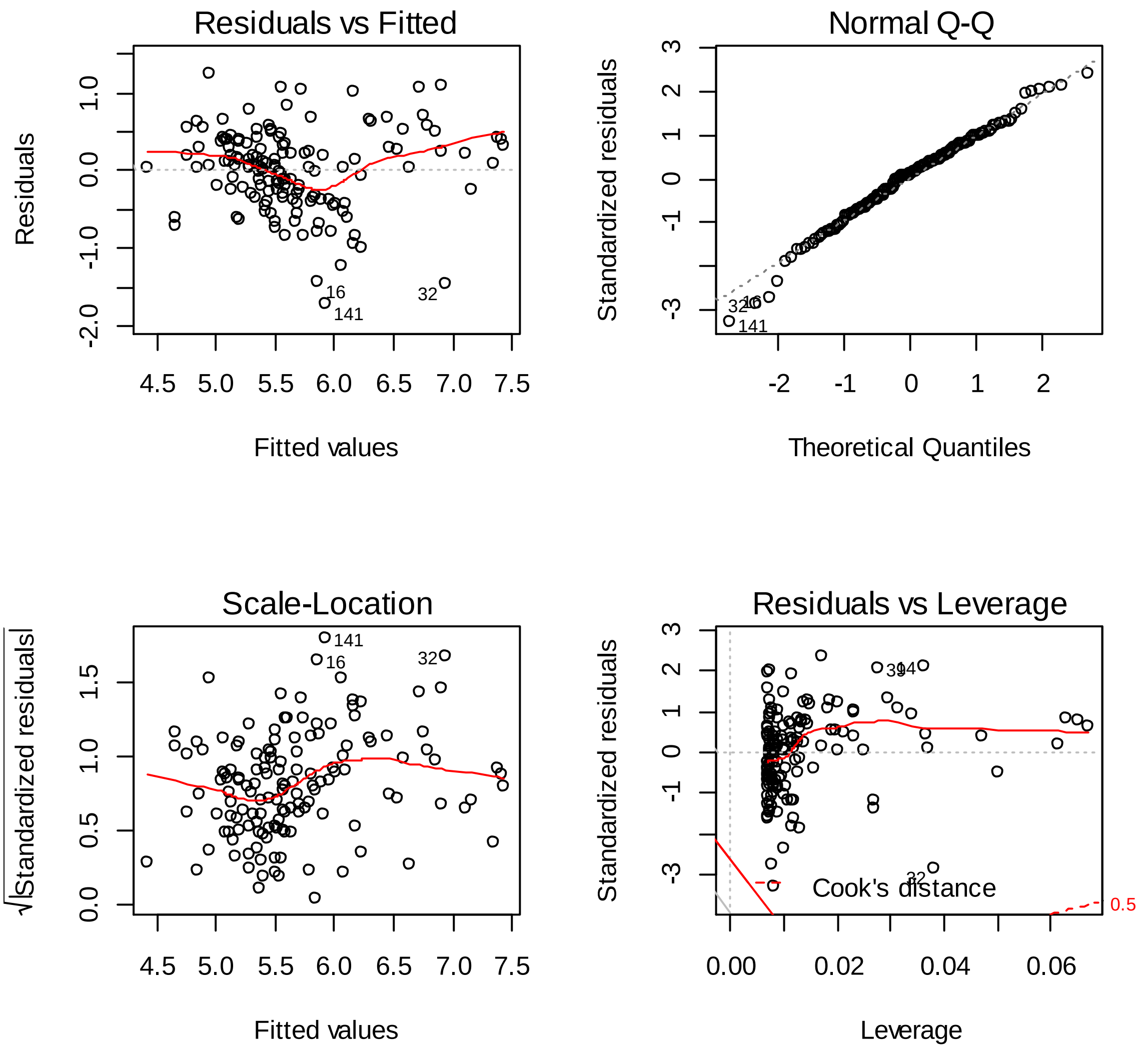

Figure S2.1: Diagnostic plots for log(APP abundance) vs. log(chlorophyll *a* concentration) fitted for all data.

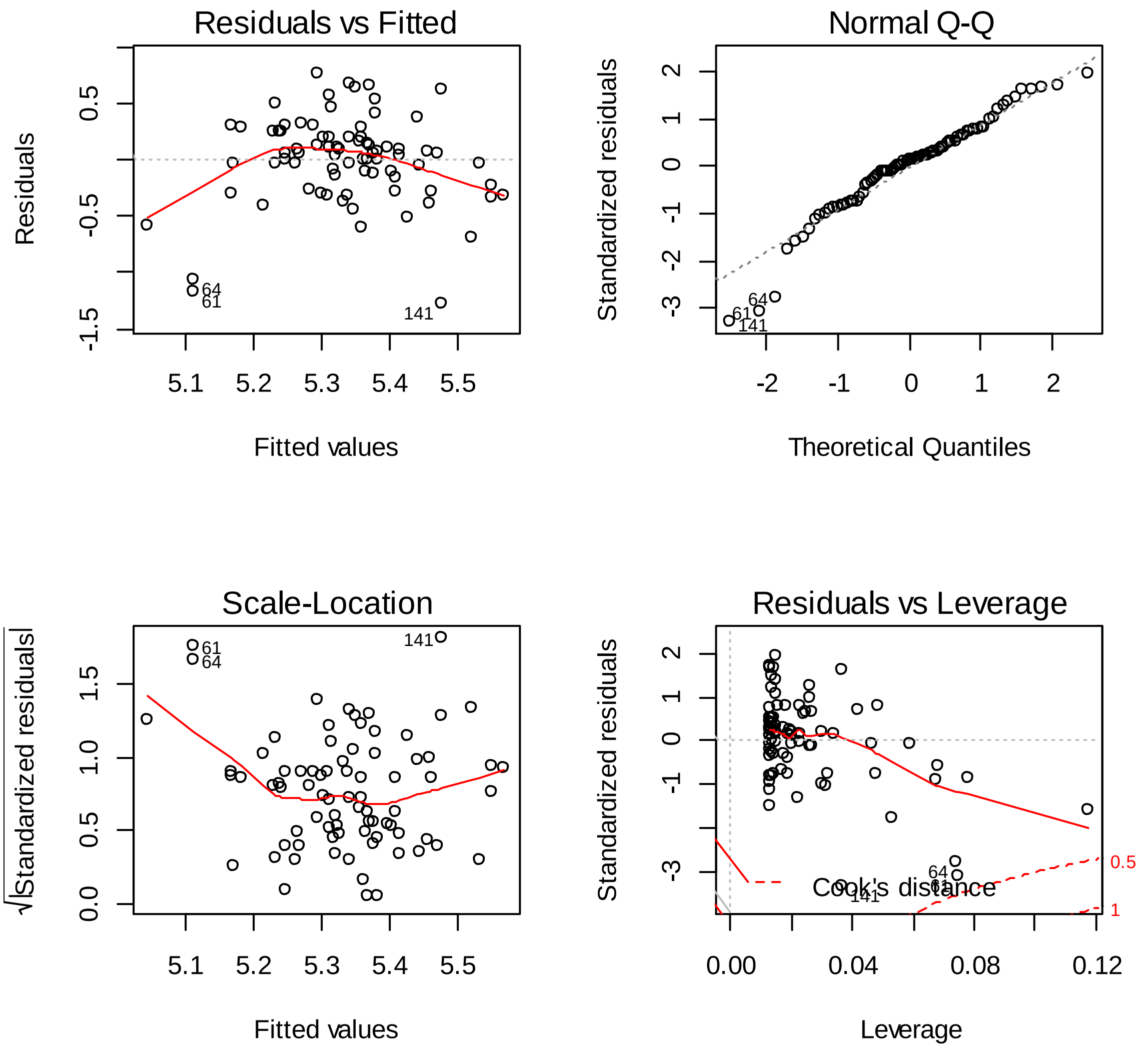

Figure S2.2: Diagnostic plots for log(APP abundance) vs. log(chlorophyll *a* concentration) fitted for data data points where organic matter free suspended solid concentration (TSS-Org) is below 50 mg/l.

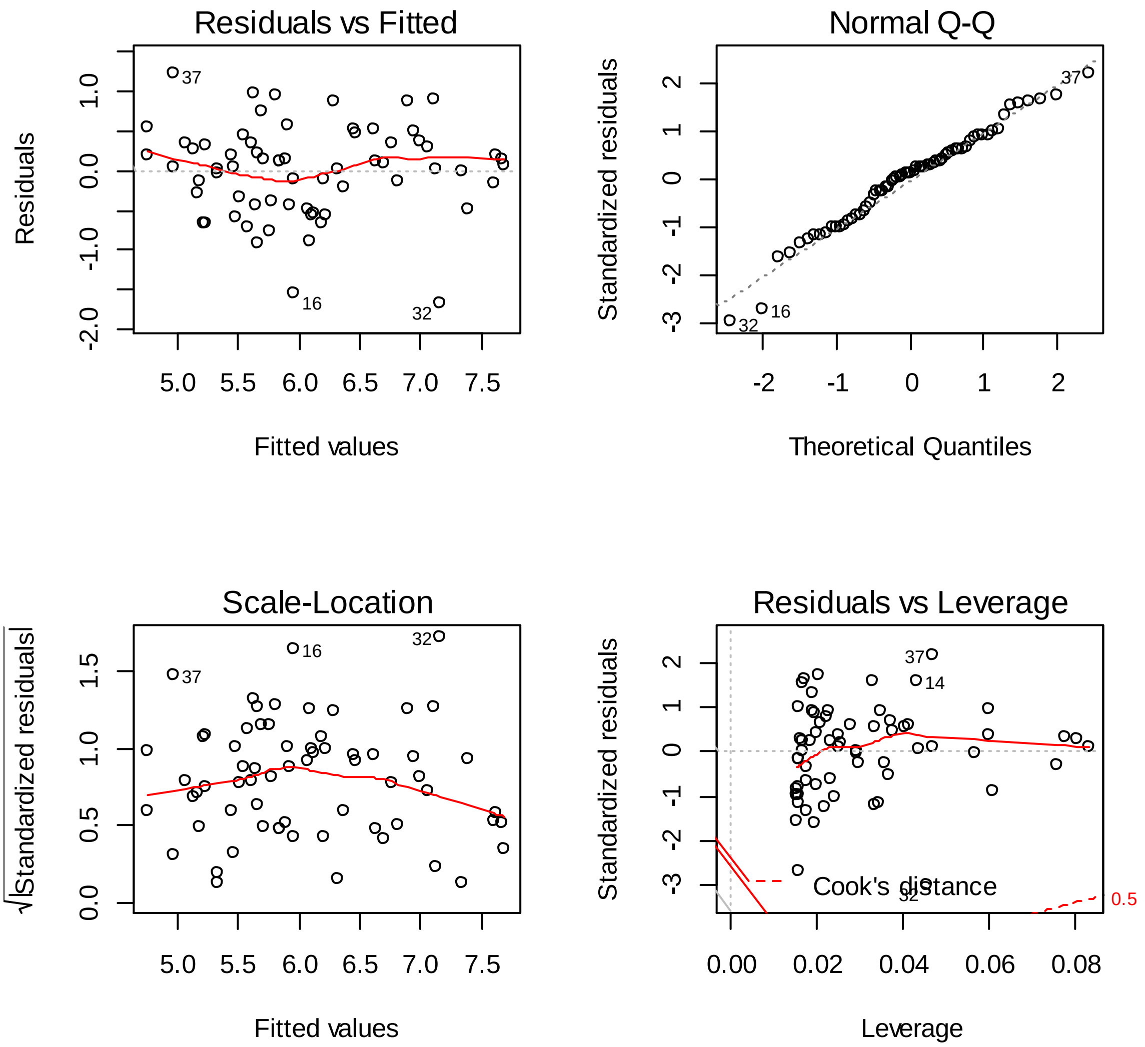

Figure S2.3: Diagnostic plots for log(APP abundance) vs. log(chlorophyll *a* concentration) fitted for data data points where organic matter free suspended solid concentration (TSS-Org) is above 50 mg/l.

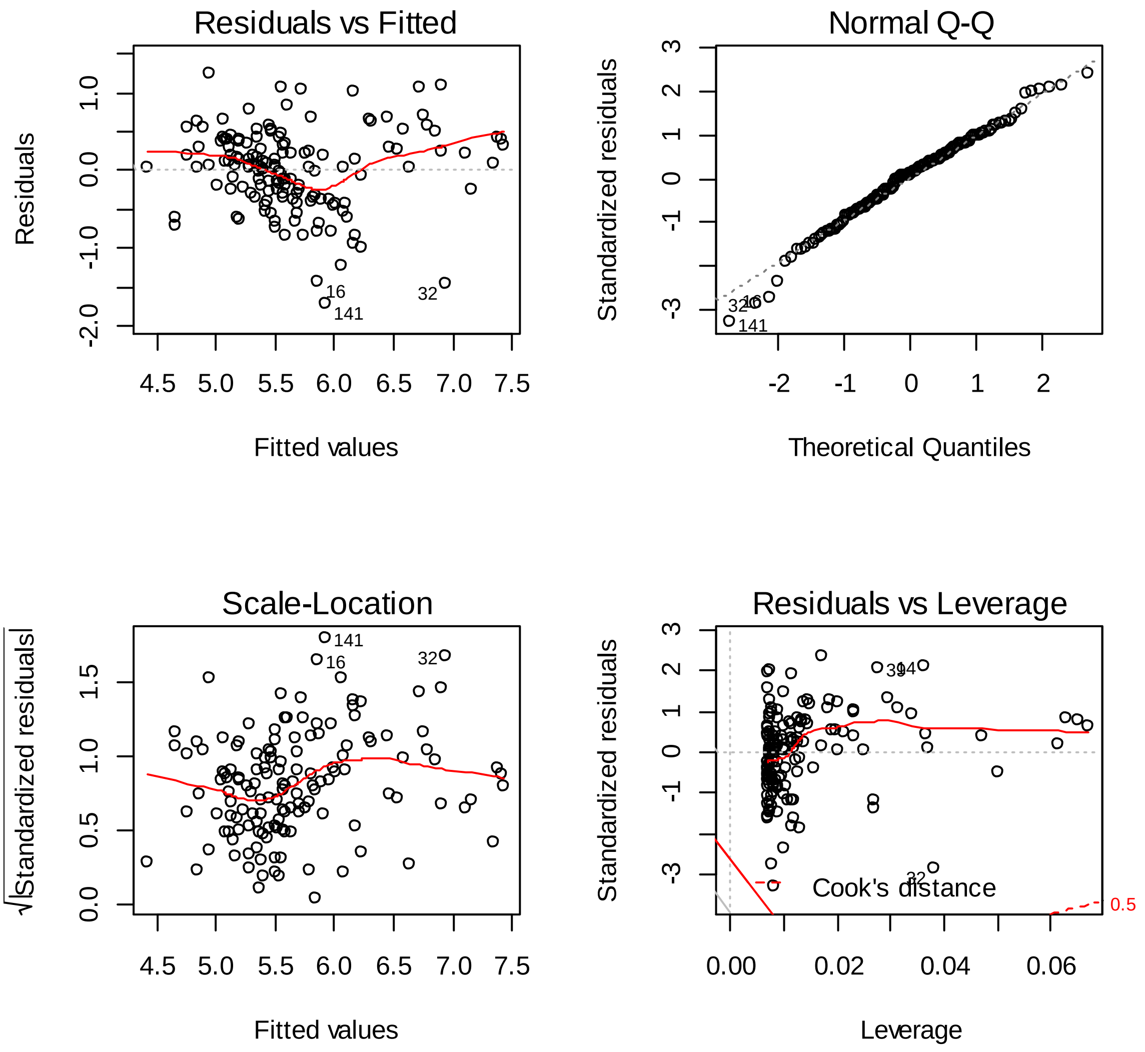

Figure S2.4: Diagnostic plots for log(APP contribution) vs. log(chlorophyll *a* concentration) fitted for all data.

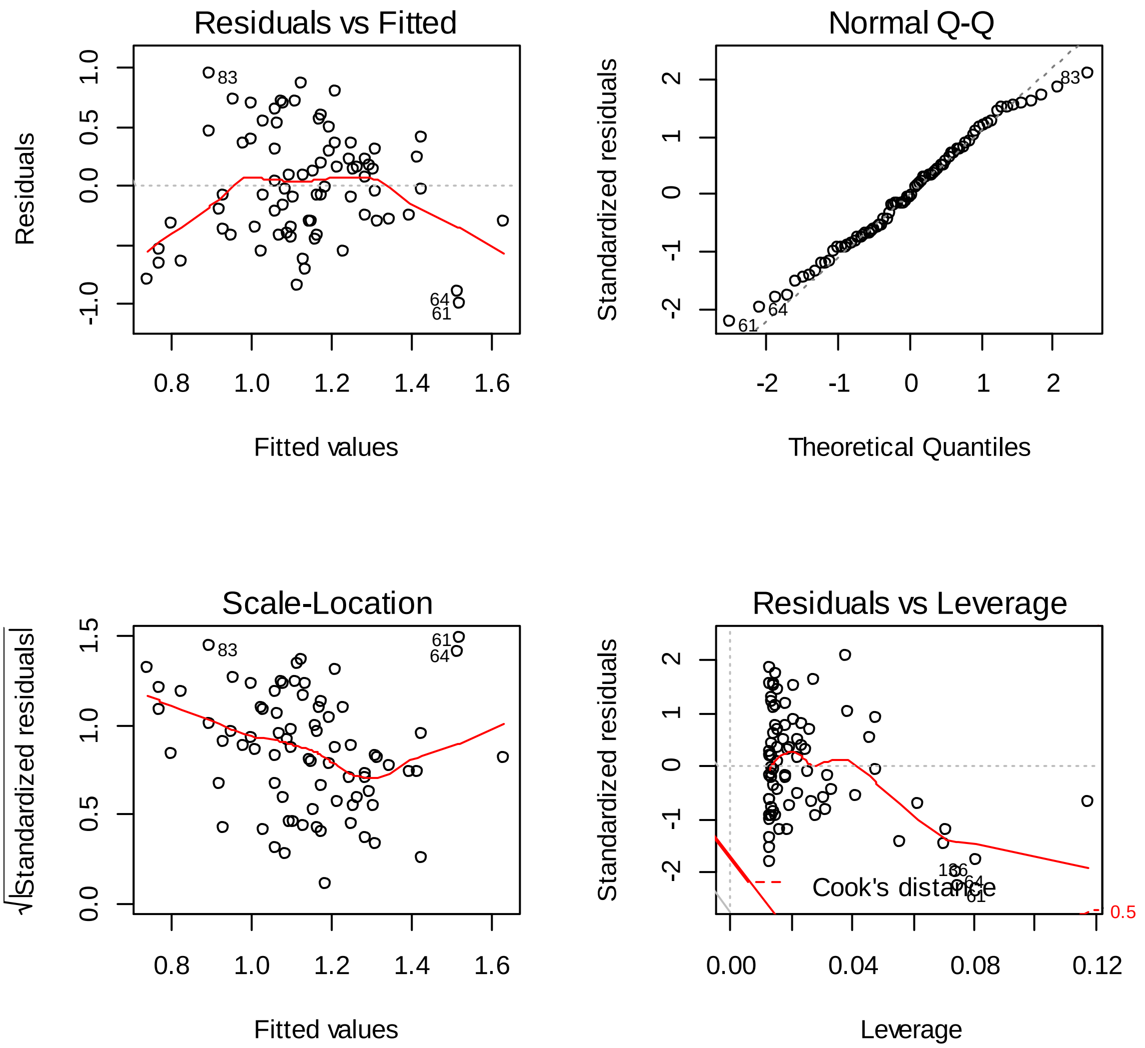

Figure S2.5: Diagnostic plots for log(APP contribution) vs. log(chlorophyll a concentration) fitted for data data points where organic matter free suspended solid concentration (TSS-Org) is below 50 mg/l.

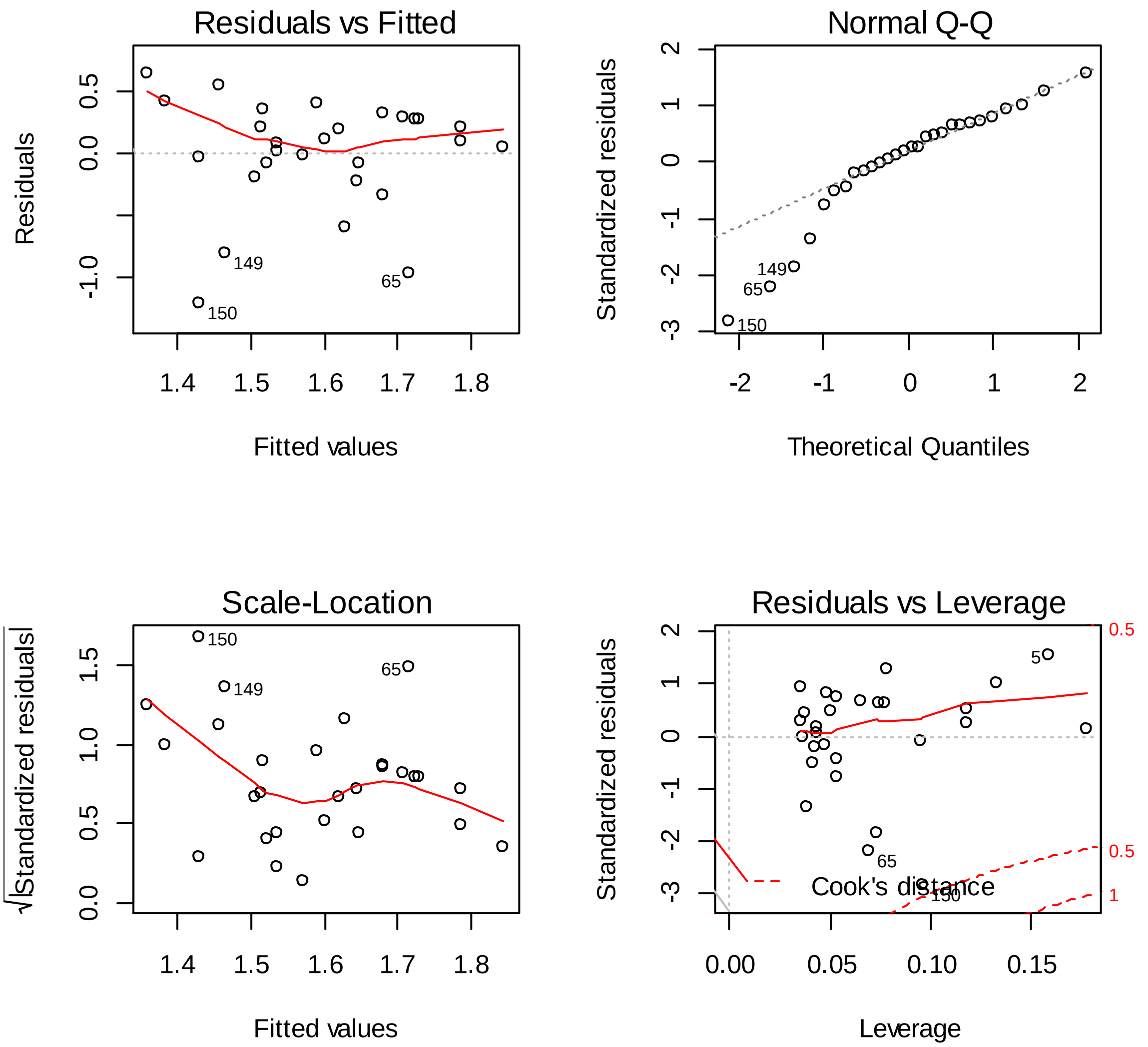

Figure S2.6: Diagnostic plots for log(APP contribution) vs. log(chlorophyll a concentration) fitted for data data points where organic matter free suspended solid concentration (TSS-Org) is between 50 and 500 mg/l.

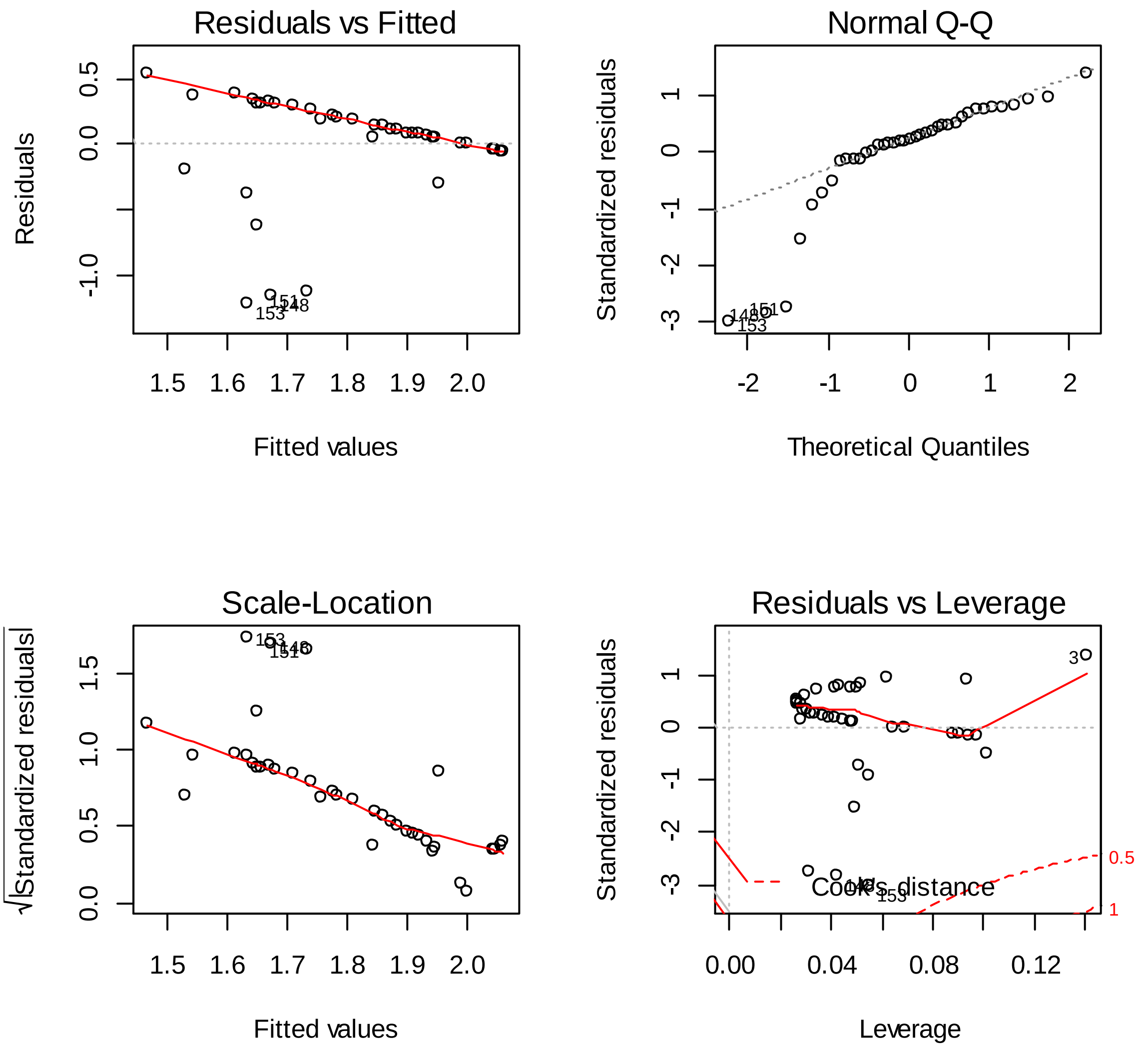

Figure S2.7: Diagnostic plots for log(APP contribution) vs. log(chlorophyll a concentration) fitted for data data points where organic matter free suspended solid concentration (TSS-Org) is between 50 and 500 mg/l.
